# miR-3574 ameliorates intermittent hypoxia-induced cardiomyocyte injury through inhibiting Axin1

**DOI:** 10.18632/aging.202480

**Published:** 2021-02-11

**Authors:** Qingshi Chen, Guofu Lin, Yongfa Chen, Chaowei Li, Lizhen Wu, Xin Hu, Qichang Lin

**Affiliations:** 1 Department of Endocrinology and Metabolism, The Second Affiliated Hospital of Fujian Medical University, Fengze, Quanzhou 362000, China; 2 Department of Respiratory and Critical Care Medicine, The First Affiliated Hospital of Fujian Medical University, Taijiang, Fuzhou 350005, China; 3 The First Affiliated Hospital of Xiamen University, Siming, Xiamen 361001, China; 4 The Second Affiliated Hospital of Fujian Medical University, Fengze, Quanzhou 362000, China

**Keywords:** miR-3574, obstructive sleep apnea, Axin1, intermittent hypoxia, cardiomyocyte injury

## Abstract

Objective: miRNAs play critical roles in the regulation of many cardiovascular diseases. However, its role and potential mechanism in cardiac injury caused by obstructive sleep apnea (OSA) remain poorly elucidated. In the present study, we aimed to investigate the effects of miR-3574 on cardiomyocyte injury under intermittent hypoxia (IH).

Results: We confirmed that IH inhibited cell viability, induced cell apoptosis and suppressed miR-3574 expression in the H9c2. miR-3574 overexpression could ameliorate the effects of IH on the cell viability and cell apoptosis in the H9c2. Axin1 was a target gene of miR-3574, and miR-3574 overexpression reduced the expression of Axin1. miR-3574 could inhibit the IH-induced cardiomyocyte injury via downregulating Axin1. However, Axin1 could partially reverse these effects of miR-3574.

Conclusion: Our study first reveals that miR-3574 could alleviate IH-induced cardiomyocyte injury by targeting Axin1, which may function as a novel and promising therapy target for OSA-associated cardiovascular diseases.

Methods: H9c2 were exposed to IH condition. CCK-8 assay was applied to determine cell viability of H9c2. qRT-PCR was conducted to measure the expression level of mRNA and miRNA. Western blot assay was then performed to detect the protein levels. Finally, we used dual-luciferase reporter assay identify the potential target of miR-3574.

## INTRODUCTION

Obstructive sleep apnea (OSA), a common chronic sleep disorder, is known to affect millions of people worldwide [1]. Intermittent hypoxia (IH) is the hallmark feature of OSA, which is likely to be the underlying cause for OSA-related comorbidities [2, 3]. Meanwhile, it is noteworthy that OSA is an independent risk for cardiovascular diseases, such as systemic arterial hypertension [4], atrial fibrillation [5], ischemic heart disease [6], metabolic disorders [4], and congestive heart failure [7]. Therefore, the understanding of the molecular mechanisms of OSA-related cardiovascular diseases and development of novel therapeutic strategies for such patients are urgently required.

microRNAs (miRNAs), a cluster of highly conserved non-coding RNAs, play a ubiquitous and key role in the regulation of gene transcription. miRNAs can modulate gene expression through binding to the 3’-UTR region of the target genes, thus resulting in either translational inhibition or degradation of the target genes [8, 9]. It has been reported that miRNAs are implicated in a wide range of cellular processes, including cell viability, development, differentiation, proliferation, and apoptosis [10, 11]. Accumulating evidence has also indicated that miRNAs played crucial roles in multiple heart diseases, such as cardiac hypertrophy [12], heart failure [13], and myocardial infarction [14]. In the past few years, miRNA expression profiles in OSA-related heart disease have been gradually revealed [15, 16]. However, the mechanism underlying the ameliorative effect of miRNAs on IH-induced H9c2 cardiomyocyte injury has not been fully elucidated.

In the current study, we established a model of IH in H9c2 cells. We found that IH could induce cardiomyocyte injury *in vitro* and lead to miR-3574 downregulation. miR-3574 upregulation ameliorated IH-induced cardiomyocyte injury through regulating cell viability and apoptosis via Axin1, indicating that miR-3574 and Axin1 can serve as a potential novel and effective therapeutic strategy for OSA-related cardiovascular diseases.

## RESULTS

### IH induces cell injury in H9c2 cardiomyocytes

**Figure 1:**
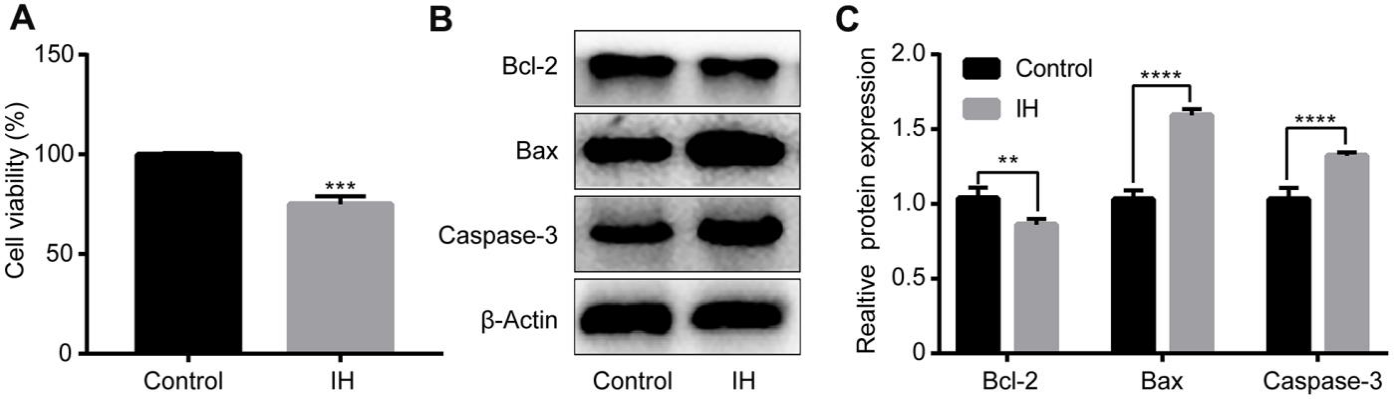
**Intermittent hypoxia induces H9c2 cell injury.** H9c2 cells were cultured under IH. (**A**) Cell viability were evaluated by CCK8 assay. (**B**, **C**) Cell apoptosis-related proteins were tested by western blotting assays. IH: Intermittent hypoxia. Data are represented as the mean ± SD from three independent experiments. * p < 0.05, ** p < 0.01.

Following IH treatment, the cell viability of H9c2 cardiomyocytes was then measured by use of CCK-8 assay. The results demonstrated that the level of cell viability was remarkably suppressed following IH treatment in the H9c2 cells (Figure 1A). Furthermore, the expression levels of certain apoptosis-associated proteins, such as Bax, Bcl-2 and caspase-3, were also detected. As shown in Figure 1B, 1C, the protein expression level of Bcl-2 was significantly downregulated, while the expression of Bax and caspase-3 were markedly upregulated following IH treatment in the H9c2 cardiomyocytes.

### IH reduces miR-3574 expression in H9c2 cardiomyocytes

**Figure 2:**
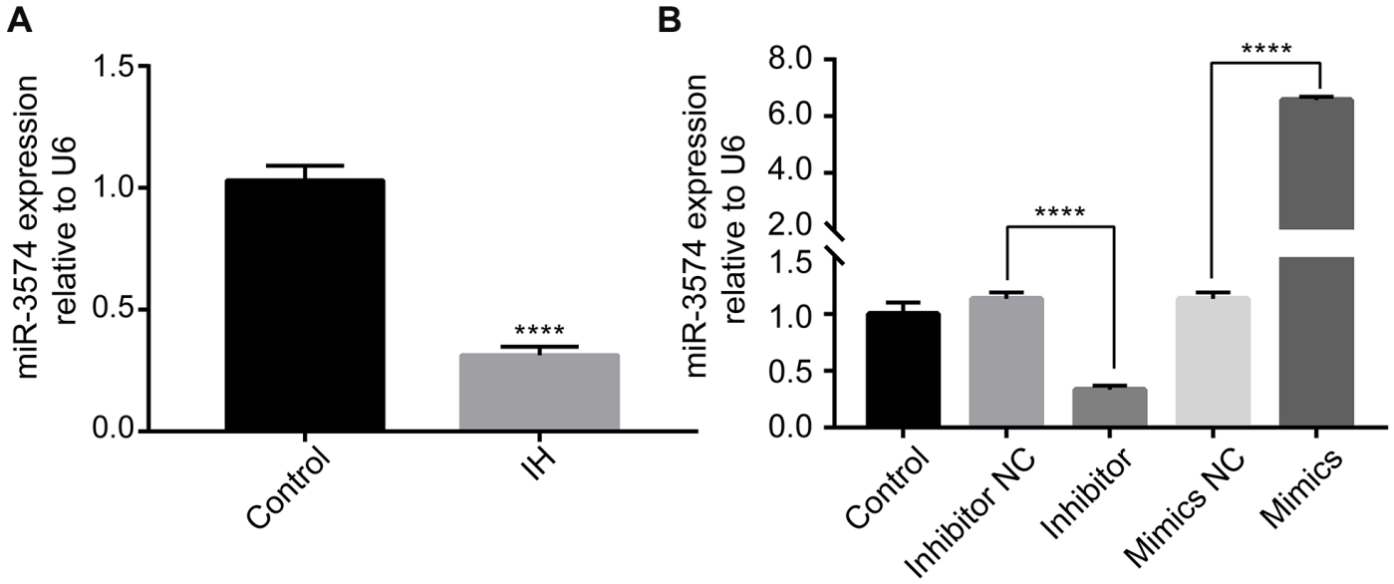
**IH reduces miR-3574 expression, and miR-3574 is differentially expressed in H9c2 cardiomyocytes after cell transfection.** (**A**) The expression of miR-3574 was detected by RT-qPCR. (**B**) Cells were transfected with miR-3574 inhibitor, miR-3574 mimics, and scrambled control. Relative miR-3574 expression was normalized to U6. IH: intermittent hypoxia. Data are represented as the mean ± SD from three independent experiments. * p < 0.05, ** p < 0.01.

miR-3574 expression in IH treated H9c2 cells was explored by RT-qPCR. The results revealed that miR-3574 expression was significantly suppressed following IH treatment of H9c2 cells (Figure 1A), indicating that IH treatment reduced the expression of miR-3574 in H9c2 cardiomyocytes. To further determine the role of miR-3574, miR-3574 was overexpressed or inhibited by transfecting with miR-3574 mimic or miR-3574 inhibitor. As shown in Figure 2B, miR-3574 mimic transfection markedly increased miR-3574 expression in the H9c2 cardiomyocytes, while miR-3574 inhibitor transfection significantly decreased miR-3574 expression in the H9c2 cardiomyocytes. These results indicated that transfection efficiency was achieved.

### Upregulation of miR-3574 attenuates IH-induced cells injury in H9c2 cardiomyocytes

**Figure 3:**
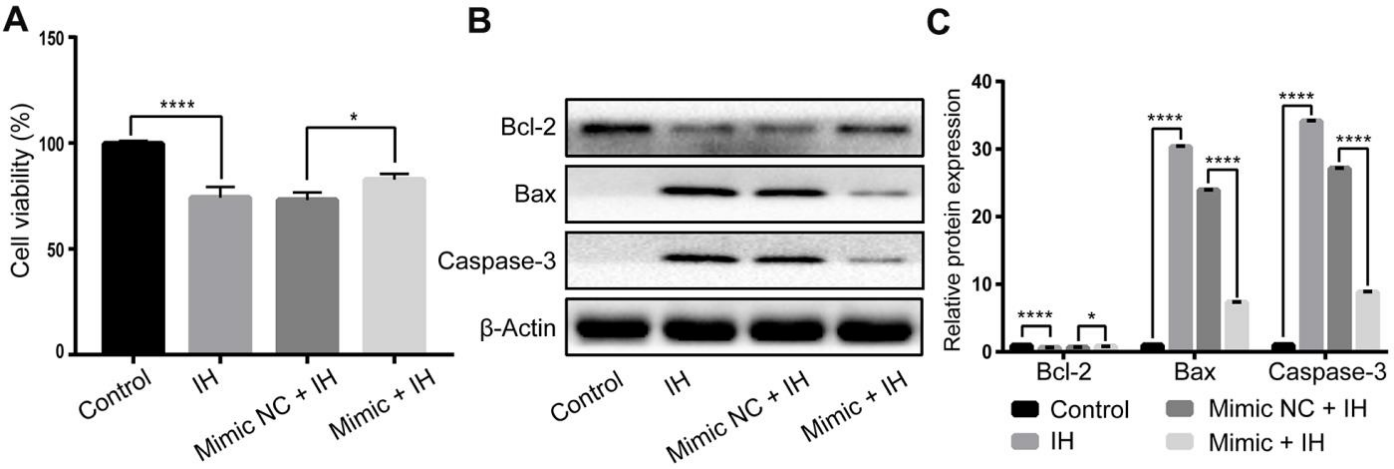
**miR-3574 attenuates IH-induced cells injury in H9c2 cardiomyocytes.** H9c2 cells were exposed to IH after transfection of miR-3574 mimics, and negative control. (**A**) Cell viability. (**B**, **C**) The expression of apoptosis-related proteins. NC: negative control. IH: intermittent hypoxia. Data are represented as the mean ± SD from three independent experiments. * p < 0.05, ** p < 0.01.

To investigate whether miR-3574 overexpression could protect the cardiomyocytes from IH-induced cells damage, H9C2 cardiomyocytes were transfected by miR-3574 mimics before IH treatment. As shown in Figure 3A, the cell viability decreased in IH-induced cardiomyocytes was increased by miR-3574 upregulation. To further determine the anti-apoptotic effects of miR-3574, the expression levels of apoptosis-associated proteins, including Bax, Bcl-2, and Caspase-3, were also examined. As we expected, western blot assay found that miR-3574 overexpression could significantly decrease the expression of Caspase-3 and Bax and increase the expression of Bcl-2 in IH-injured H9c2 cells. Altogether, these results demonstrate that miR-3574 has an anti-apoptotic role in IH-induced cardiomyocytes injury.

### miR-3574 directly targeted Axin1, and inhibited Axin1 expression

**Figure 4:**
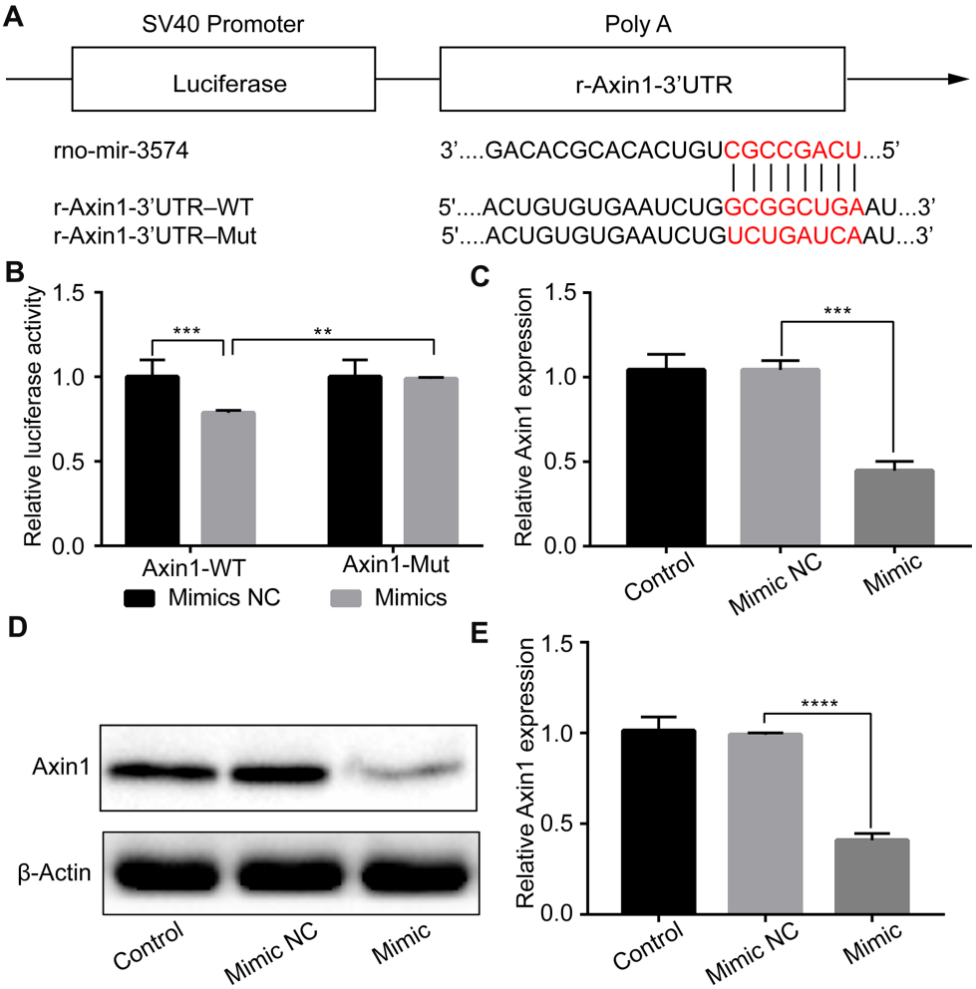
**Axin1 is a target of miR-3574, and Axin1 could be inhibited by miR-3574 in H9c2 cells.** (**A**) The potential binding site between miR-3574 and the 3’-UTR of Axin1. (**B**) Luciferase reporter assay was conducted by co-transfection of luciferase reporter containing 3’-UTR of Axin1 with miR-3574 mimic into 293T cells. (**C**–**E**) H9c2 cells were transfected with miR-3574 mimics or corresponding control. Expressions of Axin1 were examined by western blot analysis and RT-qPCR, respectively. NC: negative control. Data are represented as the mean ± SD from three independent experiments. * p < 0.05, ** p < 0.01.

Next, we sought to verify the putative targets of miR-3574 in H9c2 cells by IH treatment. We searched for the potential target genes of miR-3574 by the use of PicTar, miRanda, and TargetScan. Bioinformatics analysis identified that Axin1 may be one of the target genes (Figure 4A). To validate it, a dual-luciferase reporter assay was performed. As shown in Figure 4B, miR-3574 significantly reduced the luciferase activity of wild-type Axin1-3’-UTR, but had no effect on that of the mutant one. We then examined the regulation of miR-3574 on Axin1 expression in H9c2 cells. Western blot analysis and RT-qPCR were conducted to test the expression level of Axin1. Our results indicated that both mRNA (Figure 4C) and protein (Figure 4D, 4E) levels of Axin1 were apparently increased in H9c2 cells under IH exposure, which was remarkably suppressed by miR-3574 mimics. Therefore, our findings demonstrate that miR-3574 can directly target the 3’-UTR of Axin1 to inhibit its expression in H9c2 cardiomyocytes.

### Axin1 reverses the effects of miR-3574 overexpression on cell injury in IH-treated H9c2 cardiomyocytes

**Figure 5:**
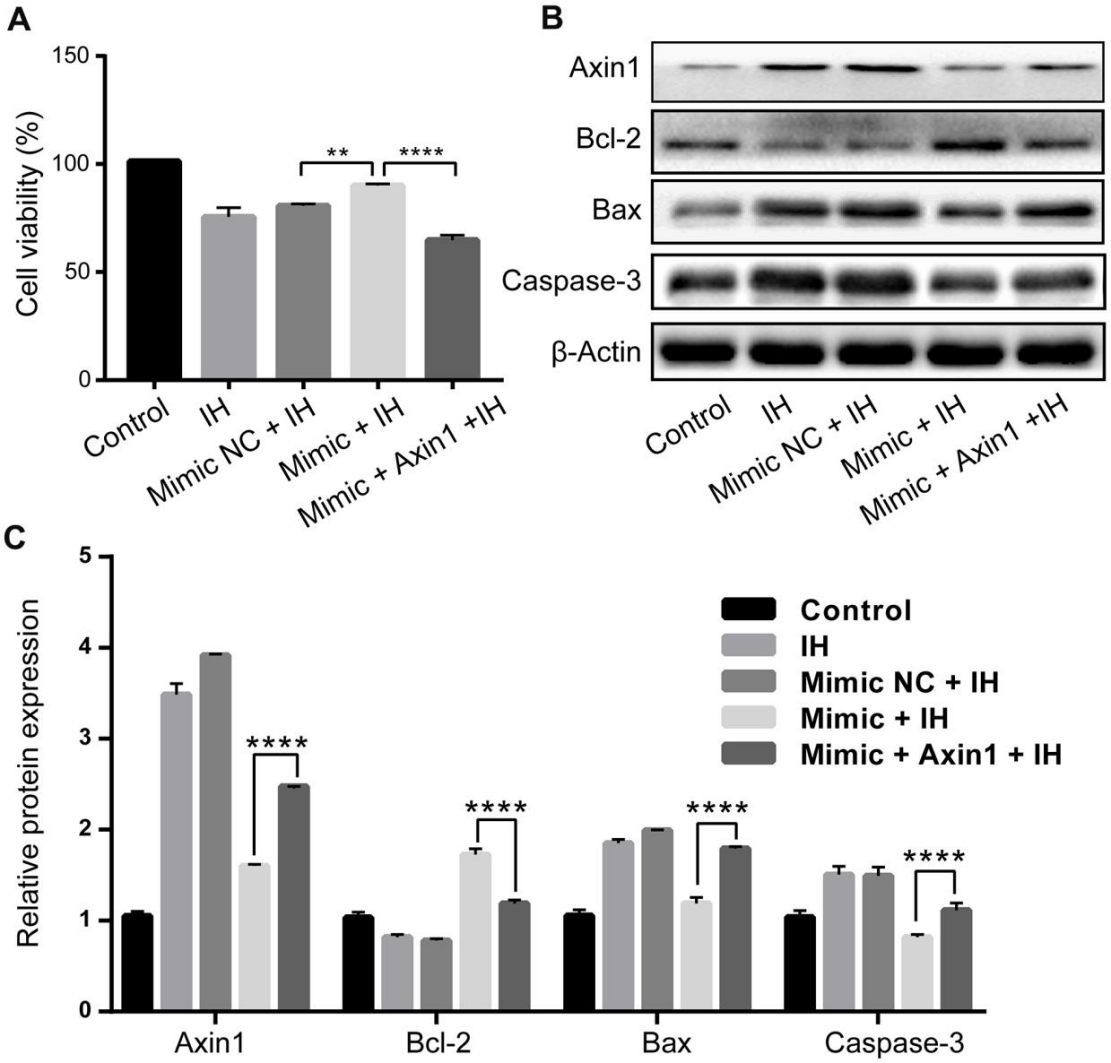
**Upregulation of miR-3574 inhibits IH-induced H9c2 cardiomyocyte injury via Axin1.** miR-3574 mimic, pcDNA3.1-Axin1 plasmid, and scrambled control were transfected into H9c2 cells. Cells without transfection were served as control. (**A**) Cell viability. (**B**, **C**) Western blot assays of Caspase-3, Bax, Bcl-2, and Axin1 protein. NC: negative control. IH: intermittent hypoxia. Data are represented as the mean ± SD from three independent experiments. * p < 0.05, ** p < 0.01.

Finally, we performed rescue experiments to further explore whether Axin1 overexpression could rescue the anti-apoptosis function of miR-3574 in IH-induced cardiomyocytes. H9c2 cells was transfected with miR-3574 mimics together without or with pcDNA3.1- Axin1 under IH condition. The results showed that the reduced level of Axin1 caused by miR-3574 overexpression could be partly rescued by co-transfection with pcDNA3.1-Axin1 (P<0.05) (Figure 5B). At the same time, functional experiments further demonstrated that the reintroduction of Axin1 could partially reverse the effects of miR-3574 on the cell viability (P<0.05) (Figure 5A) and apoptosis (P<0.05) (Figure 5B) in the IH-treated H9c2 cardiomyocytes. Taken together, our findings indicated that Axin1 acts as a downstream target of miR-3574 in the determination of cell injury in IH-treated H9c2 cardiomyocytes.

## DISCUSSION

In the present study, we found that IH treatment induced cell injury and inhibited the expression of miR-3574 in the H9c2 cardiomyocytes. miR-3574 overexpression reversed the effects of IH treatment on the cell viability and apoptosis in H9c2 cells. Using bioinformatics prediction, Axin1 was explored to be one of the potential targets of miR-3574. Next, the Luciferase reporter assay confirmed that miR-3574 suppressed the expression of Axin1 by binding with the 3’UTR of Axin1. Furthermore, it was found that miR-3574 further suppressed the mRNA and protein levels of Axin1 in the H9c2 cardiomyocytes. More importantly, enforced expression of Axin1 could partly reverse the effects of miR-3574 overexpression on cell damage in the IH treated H9c2 cardiomyocytes. Our work first revealed that miR-3574 is a critical regulator in the myocardial IH injury, which may provide a potential therapeutic target in OSA-related heart disease.

There is increased recognition that OSA, characterized by intermittent hypoxia (IH), is deemed as a risk factor for the development of many cardiovascular diseases such as hypertension [4], myocardial ischemia [6], heart failure [7], atherosclerosis [17], and coronary artery disease [18]. The IH leads to a number of potential adverse consequences, including activation of inflammation [19], insulin resistance [20], oxidative stress [21], sympathetic activation [22], and endothelial dysfunction [23]. Thus, it contributes to OSA-associated cardiovascular pathologies. For example, previous studies showed that IH could activate the factors of NF-kappaB-dependent inflammation resulting in OSA- related cardiovascular dysfunction [24]. Meanwhile, it is reported that IH-induced oxidative stress appears to involve in the hypersensitivity to myocardial infarction [25]. Additionally, our previous study observed that IH also could cause endothelial dysfunction [26], which represents one of the initial steps of atherosclerosis development. In our study, we demonstrated that IH treatment markedly decreased cell viability and altered the expression levels of apoptosis-associated proteins in H9c2 cardiac myocytes. In the recent years, how to relieve IH-related cardiomyocytes injury has attracted increasing attention.

Accumulative data indicates that a number of miRNAs have taken part in the pathogenesis of cardiovascular diseases [27–29]. Moreover, increasing evidence confirmed that a large number of miRNAs could inhibit or promote cardiomyocytes apoptosis via mediating the downstream pathways and targets. For instance, Yang et al demonstrated that miR-320 participate in the cardioprotective effect of insulin against H9c2 cardiomyocytes apoptosis by targeting survivin [30]. At the same time, Pan et al found that miR-133b-5p contributes to HPC-induced cardioprotection by the inhibition of caspase-3 and caspase-8 apoptotic signaling [31]. In 2020, Zhang and coworkers found that miR-885 could attenuate hypoxia/reoxygenation-induced cell apoptosis via modulation of AKT/mTOR signaling in human cardiomyocytes [32]. As yet, very little is known about the function of miRNAs in OSA-related cardiovascular diseases. Our study first revealed that overexpression of miR-3574 could lead to a striking increase in cardiomyocyte viability and a remarkable decrease in H9c2 cell apoptosis following exposure to IH. That is to say, miR-3574 overexpression inhibits cardiomyocytes apoptosis under IH condition. Together, all these findings suggest that miR-3574 may have a pivotal role in the progression of IH cardiomyopathy. However, the underlying mechanisms remain unknown.

To further determine the underlying mechanism of miR-3574 in H9c2 cell viability and apoptosis under IH condition, we further carried out dual-luciferase reporter assay and bioinformatic analysis. We surprisingly found that the 3’-UTR of Axin1 contained the potential conserved binding sites of miR-3574. Axin1, a cytoplasmic protein, is an important regulator of the Wnt signaling pathway via inhibition of β-catenin [33, 34]. It was reported that Axin1 is a key mediator of cell apoptosis in human melanoma cells by inhibiting the BRAFV600E [35]. Thus, we hypothesized that Axin1involved in the process of miR-3574 regulating IH-induced H9c2 cell apoptosis. Indeed, we next demonstrated that miR-3574 overexpression was related to the inhibition of luciferase activity. In addition, we also observed that miR-3574 overexpression significantly reduced the mRNA and protein levels of Axin1. At last, rescue experiments further validated that re-introduction of Axin1 in IH-treatment of H9c2 cardiomyocytes transfected with miR-3574 mimics could significantly reversed the function of miR-3574 mimics. All of above results confirmed that Axin1 was a direct target of miR-3574, which could mediate the roles of miR-3574 in H9c2 cells viability and apoptosis under IH condition.

In our study, we first showed the negative correlation of miR-3574-Axin1 pair *in vitro*, and revealed that miR-3574 could alleviate IH-induced cardiomyocyte injury via inhibition of Axin1 *in vitro*. This result highlights the important role of miRNA regulation in OSA-related cardiovascular diseases. However, the study also harbors some limitations. Firstly, the morphological change of apoptosis was not investigated, such as the use of immunofluorescence and TUNEL assay. Secondly, this study is mainly focused their attention on only *in vitro* cell-based experiments. Thus, animal experiments on miR-3574 knock in/out should be conducted in future research. Fourthly, more markers indicating cardiomyocyte injury should be provided such as LDH, cTN, cleaved caspase-3, and so on. Finally, though the sequence in miR-3574 and Axin1 3’-UTR was highly conserved among multiple species, the function of miR-3574 and its clinical applicability are still needed to be further explored.

## CONCLUSIONS

In conclusion, we first confirmed that miR-3574 overexpression inhibited IH-induced apoptosis in cardiomyocytes by targeting Axin1. Our findings identify that miR-3574 exerts a prominent cardioprotective effect on IH-induced cardiomyocyte injury, which seems to provide a novel target for treating OSA-related heart diseases.

## MATERIALS AND METHODS

### Cell culture

H9c2 cardiomyocytes were obtained from Chinese Academy of Sciences Shanghai Cell Bank. Cells were routinely cultured in DMEM (HyClone) containing 10% fetal bovine serum (Gibco) in a 5% CO_2_ humidified incubator at 37° C.

### IH treatment

When H9c2 cardiomyocytes reached 70-80% confluence, IH stimulation was carried out according to the method as we previously described [36]. Briefly, cells were first maintained under hypoxia for 35 min, which was induced by flushing a mixed air of 5% CO_2_, 1% O_2_ and 94% N_2_. Subsequently, cells were then cultured under normoxia condition for 25 min (5% CO_2_, 21% O_2_ balanced with N_2_). Repeated IH treatment was conducted for 6 cycles.

### RNA isolation and qRT-PCR

Total RNA was extracted from H9c2 cardiomyocytes by use of the Trizol Reagent (Invitrogen, USA). The quality was determined by gel electrophoresis and NanoDrop 2000 (Thermo Fisher Scientific, USA). According to the manufacturers’ protocols, the reverse transcription of miRNA and mRNA from the total RNA was employed by using Mir-X™ miRNA First Strand Synthesis Kit (Clontech, USA) and PrimeScript™ RT Reagent Kit (Takara, China), respectively. qRT-PCR reactions were prepared by using SYBR Green PCR Master Mix (Thermo Fisher Scientific) and performed in ABI 7500 real-time RT-PCR system. β-actin and U6 were used as internal controls. All primers used in this study were listed in Table 1. Relative expression levels of miRNA and mRNA was calculated using the 2^−∆∆Ct^ method. All experiments were conducted in triplicate.

**Table 1:** Primers used for RT-qPCR.

**ID**	**Sequence (5′-3′)**
miR-3574	Sense: CATCAGCCGCTGTCACACG
	Antisense: CCAGTGCGTGTCGTGGAGT
miR-3574 mimic	Sense: UCAGCCGCUGUCACACGCACAG
	Antisense: CUGUGCGUGUGACAGCGGCUGA
miR-3574 mimic NC	Sense: UUGUACUACACAAAAGUACUG
	Antisense: GUACUUUUGUGUAGUACAAUU
miR-3574 inhibitor	CUGUGCGUGUGACAGCGGCUGA
miR-3574 inhibitor NC	CAG UAC UUU UGU GUA GUA CAA
Axin1	Sense: GAAGACGGCGATCCATCG
	Antisense: GGATGCTCTCAGGGTTCT
U6	Sense: CTCGCTTCGGCAGCACA
	Antisense: AACGCTTCACGAATTTGCGT
β-Actin	Sense: CGAGTACAACCTTCTTGCAGC
	Antisense: ACCCATACCCACCATCACAC

### Cell counting kit-8 (CCK-8) assay

CCK-8 assay was performed to evaluate cell viability. H9c2 cells were maintained in 96-well plates and gained the relevant treatments, including IH, transfection. For CCK8 detection, 10 μl/well of CCK-8 solution was added into each well. The absorbance was determined with the use of a microplate reader (Bio-Rad) at 450 nm.

### Western blot analysis

Total proteins were first extracted from H9c2 cardiomyocytes using RIPA buffer (Beyotime, China). We used the BCA Protein Assay Kit (Thermo Scientific, USA) to detect the protein concentration. Subsequently, we separated equal amounts of protein on 10% SDS-PAGE, and then transferred the proteins to a PVDF membrane. After blocking with 5% nonfat milk, we then incubated the membrane with appropriate primary antibodies at 4° C overnight. Next, we employed TBST to wash the membrane three times. Then, it was incubated with secondary antibodies conjugated with horseradish peroxidase (Santa Cruz Biotechnology) for 1 h at room temperature. At last, the signals were visualized on X-ray films by using enhanced chemiluminescence (ECL). The bands were analyzed with the use of ImageJ software. The expression level of relative proteins was normalized to β-Actin.

### Cell transfection

H9c2 cardiomyocytes were detached with trypsin (Gibco) and counted the day before transfection. H9c2 cardiomyocytes were cultured to reach 90% confluence on the day of transfection. The miR-3574 inhibitor and mimic, and scrambled control were obtained from Sangon Biotech Co. (Shanghai, China), which were listed in Table 1. At the same time, the pcDNA3.1-Axin1 plasmid and vector were also obtained from Sangon Biotech Co. Then, we transfected miRNAs and pcDNA3.1 plasmids into H9c2 cells with Lipofectamine 3000 (Invitrogen, USA). All transfections were carried out before the IH treatment, and then cultured for 48 h for subsequent experimentation.

### Dual-luciferase reporter assay

We generated the mutant 3’-UTR of Axin1 by use of the Quick-Change Site-Directed Mutagenesis kit (Stratagene, USA). The 3’-UTR of Axin1 contained either the miR-3574 binding site or the mutant 3’-UTR of Axin1, which was cloned into the psiCHECK-2 vectors (Promega, USA) to synthesize the psiCHECK-Axin1 3’-UTR Luciferase reporter plasmid. Following the manufacturer’s instruction, luciferase reporter plasmids and miR-3574 mimic or miR-NC were then co-transfected into H9c2 with the help of Lipofectamine 3000 reagent (Invitrogen, USA). After 48 h, we applied a Dual-Luciferase reporter gene assay kit (Promega, USA) to detect the Luciferase activities. Renilla luciferase was served as normalisation. The experiment was repeated three times.

### Statistical analysis

All data were displayed as the mean ± SD from at least three independent experiments. GraphPad Prism 7 and SPSS 22.0 were used to analyze the data. We analyzed the differences between two independent groups by using Student’s t tests. One-way ANOVA were used for the comparison of differences among multiple groups. P < 0.05 was deemed statistically significant.
